# Draft Genome of Australian Environmental Strain WM 09.24 of the Opportunistic Human Pathogen Scedosporium aurantiacum

**DOI:** 10.1128/genomeA.01526-14

**Published:** 2015-02-12

**Authors:** Åsa Pérez-Bercoff, Alexie Papanicolaou, Marc Ramsperger, Jashanpreet Kaur, Hardip R. Patel, Azian Harun, Shu Yao Duan, Liam Elbourne, Jean-Philippe Bouchara, Ian T. Paulsen, Helena Nevalainen, Wieland Meyer, Gavin A. Huttley

**Affiliations:** a Centre for Infectious Diseases and Microbiology, Molecular Mycology Research Laboratory, Marie Bashir Institute for Infectious Diseases and Biosecurity, Sydney Medical School-Westmead Hospital, University of Sydney, Westmead Millennium Institute, Sydney, NSW, Australia; b John Curtin School of Medical Research, Australian National University, Arts and Culture Trust, Canberra, Australia; c CSIRO Land and Water Flagship, Black Mountain Laboratories, CSIRO, Arts and Culture Trust, Canberra, Australia; d Hawkesbury Institute for the Environment, University of Western Sydney, Arts and Culture Trust, Penrith, Australia; e Chemistry and Biomolecular Sciences, Macquarie University, Sydney, NSW, Australia; f School of Medical Sciences, Universiti Sains Malaysia, Kota Bharu, Kelantan, Malaysia; g L’UNAM Université, Université d’Angers, Groupe d'Etude des Interactions Hôte-Pathogène, Angers, France; h Laboratoire de Parasitologie-Mycologie, Centre Hospitalier Universitaire, Angers, France

## Abstract

We report here the first genome assembly and annotation of the human-pathogenic fungus Scedosporium aurantiacum, with a predicted 10,525 genes, and 11,661 transcripts. The strain WM 09.24 was isolated from the environment at Circular Quay, Sydney, New South Wales, Australia.

## GENOME ANNOUNCEMENT

Scedosporium aurantiacum is a hyphomycetous filamentous fungus found in various environments such as soil, sewage and polluted waters (1). It is an emerging opportunistic pathogen capable of causing a range of infections that are especially abundant in Australia (2). To enable the genetic characterization of the virulence and high antifungal resistance potential of this species (3), the genome of the environmental, highly virulent S. aurantiacum strain WM 09.24, collected from Circular Quay, Sydney, NSW, Australia, in 2009, was sequenced (A. Harun and W. Meyer, unpublished data). The isolate selection was based on a global multilocus sequence typing (MLST) study (A. Harun and W. Meyer, unpublished data) and determination of its virulence using the Galleria mellonella model (S. Duan and W. Meyer, unpublished data).

Genomic DNA was sequenced (paired-end [PE] reads) on Illumina HiSeq 2000 at the Ramaciotti Centre for Genomics, University of New South Wales (UNSW), Sydney, NSW, Australia. Trimmomatic (version 0.27) (4) was used to clip off adapters and trim the reads prior to assembly with SPAdes (version 3.1.1) (5). After correction with REAPR (version 1.0.16) (6), and discarding of scaffolds <200 nucleotides (nt), the assembly consisted of 1,584 scaffolds (202 nt to 380,183 nt in length) and had a total genome size of 39,890,731 nt with 49.20% GC content. The assembly’s *N*_50_ was 78,269 nt and *N*_90_ was 16,521 nt and was calculated to have an average coverage of 162× over all 1,584 contigs. The CEGMA pipeline (7) indicated an assembly completeness of 93.15%. RepeatMasker version open-4.0.3 (http://www.repeatmasker.org) was used to identify repeats in the genome assembly and 1.96% of the bases were masked.

To facilitate genome annotation, RNA-seq was obtained from S. aurantiacum strain WM 09.24 grown in different media at 25°C and 37°C, pertaining to environmental and human host growth conditions, respectively, as follows: (i) water medium for starvation, (ii) potato dextrose (PD) to promote good general growth, and (iii) artificial sputum medium (ASM) to simulate growth conditions in an infected lung of a cystic fibrosis (CF) patient (8; M. Ramsperger and W. Meyer, unpublished data). Single-end (SE) reads of 100 bp were generated using the Illumina HiSeq 2500 at the Biomolecular Research Facility (BRF) at the John Curtin School for Medical Research (JCSMR), Australian National University (ANU), Canberra, ACT, Australia. Genome annotation was performed using the JAMg pipeline (9) and GMAP (version 2014-12-06) (10). We predicted 10,525 gene models and 11,661 transcripts for the genome assembly of S. aurantiacum strain WM 09.24. The number of inferred genes in S. aurantiacum is comparable to Trichoderma virens, which has 12,400 genes (11), and to the recently published genome of Scedosporium apiospermum strain IHEM 14462, which was found to have 10,919 coding sequences (CDSs) (12).

The herein-reported high-quality draft assembly of the environmental and highly virulent S. aurantiacum strain WM 09.24, together with the recently published genome of S. apiospermum strain IHEM 14462 (12) will provide fundamental genomics resources to study the genetic basis of virulence and antifungal resistance in this genus.

### Nucleotide sequence accession numbers.

This whole genome shotgun project has been deposited at DDBJ/EMBL/GenBank under the accession number JUDQ00000000. The version described in this paper is the first version, JUDQ01000000.
